# The American Year-Book of Medicine and Surgery

**Published:** 1898-05

**Authors:** 


THE AMERICAN YEAR-BOOK OF MEDICINE AND SURGERY. Being a Yearly Digest of Scientific Progress and Authoritative Opinion in all branches of Medicine and Surgery, drawn from Journals, Monographs and Text-Books of the Leading American and Foreign Authors and Investigators. Collected and arranged with critical editorial comments. By Samuel W. Abott, M. D.; John J. Abel, M. D.; J. M. Baldy, M. D.; Chas. H. Burnett, M. D.; Archibald Church, M. D.; J. Chalmers DaCosta, M. D.; W. A. Newman Dorland, M. D.; Louis A. Duhring, M. D.; Virgil P. Gibney, M. D.; Homer W. Gibney, M. D.; Henry A. Griffin, M. D.; Jno. Guiteras, M. D.; Howard F. Hansel, M. D.; Burton Cooke Hirst, M. D.; E. Fletcher Ingalls, M. D.; Wyatt Johnson, M. D.; W. W. Keen, M. D.; Henry G. Ohls, M. D.; William Pepper, M. D.; Wendell Reber, M. D.; David Riesman, M. D.; Louis Starr, M. D.; Alfred Stengel, M. D.; G. N. Stewart, M. D.; C. A. Homann, M. D.; J. R. Tillinghast, M. D.; Thompson S. Westcott, M. D. Under the general editorial charge of George M. Gould, M. D. Illustrated. Published by W. B. Saunders, 925 Walnut St., Philadelphia. Price, cloth, $6.50; morocco, $7.50. For sale by subscription.

The American Year-Book for 1898 would be hard to improve upon in any respect. While not too voluminous for the perusal of the busiest practitioner, it contains sufficient space for an accurate resume of all the important work accomplished during the year. A perusal of the names of its twenty-seven editors, to say nothing of its distinguished editor in chief, is a sufficient guarantee that the work is done to the highest grade of excellence.

To those who have not had the advantage of reading former issues of this work, we will state, that it is without doubt the best work of the kind published. The present volume contains 1020 pages of text, on several hundred distinct subjects. An abstract is given of the most important and authoritative literature of each subject, and in the same connection is the editorial comment, which contains whatever information that may not have appeared in the abstract, and many well-seasoned and timely comments of the editor himself.

When it is stated that the department of General Medicine is written by Prof. William Pepper and Alfred Engel, that of General Surgery by W. W. Keen and J. Chalmers DaCosta, that of Obstetrics by Barton Cooke Hirst, and that of Pediatrics by Louis N. Starr, no doubt can be entertained of the high order of the work. The other departments are equally well cared for.

Doctor, you have no doubt read many scraps of information about the progress of the antitoxin treatment for diphtheria, of Sanarelli's bacillus icteroides, of Widal's serum test for the diagnosis of typhoid, of the many new operative procedures in apendicitis and hernia, and have a confused, undigested recollection of the mass of information, now rapidly fading from your memory. Here you have it all carefully preserved that is worth preserving, carefully digested and arranged, covered with a most elaborate and convenient index of 54 pages, and well printed and bound for preservation and future reference.

